# Longitudinal change in SARS-CoV-2 seroprevalence in 3-to 16-year-old children: The Augsburg Plus study

**DOI:** 10.1371/journal.pone.0272874

**Published:** 2022-08-11

**Authors:** Vincenza Leone, Christa Meisinger, Selin Temizel, Elisabeth Kling, Michael Gerstlauer, Michael C. Frühwald, Katrin Burkhardt

**Affiliations:** 1 Chair of Epidemiology, University of Augsburg, University Hospital Augsburg, Augsburg, Germany; 2 Department of Hygiene and Environmental Medicine, University Hospital Augsburg, Augsburg, Germany; 3 Institute of Laboratory Medicine and Microbiology University Hospital Augsburg, Augsburg, Germany; 4 Paediatric and Adolescent Medicine University Hospital Augsburg, Augsburg, Germany; Waseda University: Waseda Daigaku, JAPAN

## Abstract

**Background:**

Currently, more than 30,200,000 COVID-19 cases have been diagnosed in Germany alone. However, data regarding prevalence of COVID-19 in children, both in Germany and internationally, are sparse. We sought to evaluate the number of infected children by measuring IgG antibodies.

**Methods:**

Oropharyngeal swabs were collected between December 2020 and August 2021 to measure SARS-CoV-2, and capillary blood for the detection of SARS-CoV-2 antibodies (by rapid test NADAL® and filter paper test Euroimmun® ELISA); venous blood was taken for validation (Roche® ECLIA and *recom*Line Blot) in 365 German children aged 3–16 years from 30 schools and preschools. We used multiple serological tests because the filter paper test Euroimmun® ELISA performs better in terms of sensitivity and specificity than the rapid test NADAL®. The Roche® ECLIA test is used to detect SARS-CoV-2 spike protein, and the recomLine Blot test is used to rule out the possibility of infection by seasonal SARS-viruses and to test for specific SARS-CoV-2 proteins (NP, RBD and S1). In addition, one parent each (n = 336), and 4–5 teachers/caregivers (n = 90) per institution were tested for IgG antibodies from capillary blood samples. The total study duration was 4 months per child, including the first follow-up after 2 months and the second after 4 months.

**Results:**

Of 364 children tested at baseline, 3.6% (n = 13) were positive for SARS-CoV-2 IgG antibodies using Euroimmun® ELISA. Seven children reported previously testing positive for SARS-CoV-2; each of these was confirmed by the Roche® Anti-SARS-CoV-2-ECLIA (antibody to spike protein 1) test. SARS-CoV-2 IgG antibodies persisted over a 4-month period, but levels decreased significantly (p = 0.004) within this timeframe. The median IgG values were 192.0 BAU/ml [127.2; 288.2], 123.6 BAU/ml [76.6; 187.7] and 89.9 BAU/ml [57.4; 144.2] at baseline, 2 months and 4 months after baseline, respectively. During the study period, no child tested positive for SARS-CoV-2 by oropharyngeal swab. A total of 4.3% of all parents and 3.7% of teachers/caregivers tested positive for IgG antibodies by Euroimmun® ELISA at baseline.

**Conclusion:**

We noted a rather low seroprevalence in children despite an under-reporting of SARS-CoV-2 infections. Measurement of IgG antibodies derived from capillary blood appears to be a valid tool to detect asymptomatic infections in children. However, no asymptomatic active infection was detected during the study period of 4 months in the whole cohort. Further data on SARS-CoV-2 infections in children are needed, especially in the group of <5-year-olds, as there is currently no licensed vaccine for this age group in Germany. The Robert Koch Institute’s Standing Commission on Vaccination (STIKO) recommended COVID-19 vaccination for 12–17 and 5–11 year olds in August 2021 and May 2022 respectively.

## Introduction

Severe acute respiratory syndrome coronavirus 2 (SARS-CoV-2) is responsible for the coronavirus disease 2019 (COVID-19), which has caused more than 570,000,000 cases of infection as well as more than 6,300,000 deaths worldwide until now (July 2022) [1]. In Germany, more than 30,200,000 infections [2] and more than 143,000 deaths [3] have been recorded since the occurrence of the first case of SARS-CoV-2 infection in January 2020 [4]. Regarding SARS-CoV-2 infections in children, Ludvigsson et al. conducted a systematic review (including studies that had been published by 03/18/2020) and concluded that the proportion of children diagnosed with COVID-19 is about 1–5%. Most of the studies considered in this review were from China and some child-related data from Italy, Iran, or South Korea [2,4,5]. In Germany, among 0- to 14-year-old children, over 5,000,000 cases of infection (incidence = 154,600/ 100,000) were reported to the Robert Koch-Institute (RKI) by August 2021, with no deaths in this group [2].

Sero-epidemiological studies have been initiated to collect more detailed data regarding infection events in children. In these studies, SARS-CoV-2-specific IgG antibodies were measured. This is particularly useful because children suffering from COVID-19 are mostly asymptomatic or only mildly infected, drawing little medical attention in comparison to adults [5–7]. As a result, children are tested less frequently for SARS-CoV-2. This could potentially result in an underestimation of the true infection rate in children. It also makes it difficult to answer the question of whether children are drivers of the pandemic or not, as they may spread the virus to a greater amount than adults for a variety of reasons [8].

So far, particularly in children, data is not sufficient to determine how long SARS-CoV-2 antibodies may be detectable in blood. New data from southwest Germany demonstrate that 33.8% of a cohort of SARS-CoV-2 exposed children, aged 6–13 years, were seropositive and that 96.2% of them remained seropositive 11–12 months post infection [9].

Since the current data is inconclusive, more information is required on seroprevalence in children and adolescents considering the COVID-19 pandemic is still ongoing. This may provide a better basis for decision-making on measures for childcare centers and schools to further control the COVID-19 pandemic.

To address this issue, we performed the prospective “Augsburg Plus”-Study at the University Hospital Augsburg. The aim of the study was to address the following objectives: 1) To determine the level of antibody immune response to SARS-CoV-2 among preschool- and school-children (age-range 3–16) as well as parents, teachers, and educators 2). To evaluate the infection dynamic over a period of six months.

## Methods

### Study design

This was a prospective study on the infection dynamics of SARS-CoV-2 infections in children conducted by testing for SARS-CoV-2-transcription-mediated amplification (TMA) and SARS-CoV-2 IgG antibodies in serum over several points in time. The study was conducted at the University Hospital Augsburg in Bavaria (Germany). Altogether 18 childcare facilities, 6 elementary schools, 4 secondary schools and 2 schools with both school types (elementary and secondary school)—a total of 30 facilities within the district and city of Augsburg—cooperated with the Augsburg Plus Study. The institutions were recruited via an initial letter from the Augsburg school authorities for primary and secondary schools, diocese and the district office responsible for preschools, respectively. Since there was initially an insufficient response from the institutions, the sponsors and facility managers were again contacted by phone or e-mail to ask them to collaborate. Children who attended one of the collaborating facilities were enrolled voluntarily and randomly in the study if their parents agreed. The investigations proceeded from December 2020 to August 2021. It was planned to invite each participant to three examinations (baseline visit, first follow-up after 4 months, second follow-up after 6 months). In the end, the actual follow-up intervals between appointments were shorter than planned due to an initial low level of enrollments, and were a median length of 2 months each.

Children from the collaborating institutions aged 3–16 years and one of their parents were included in the study. Parents had no limitation in age. The aim was to screen 40 children per facility. In addition, about 4 educators or teachers from each of the institutions could register for participation. Each participant had to agree in writing to voluntary participation. An additional consent form was prepared for the children who could already read, in order to inform the children as best as possible. The written informed consent forms for the children were administered by the parents. Each participant was informed that the examinations could only take place if the participants were free from COVID-19 symptoms. If this was the case, the appointed examination date was scheduled. This was applied to each examination appointment.

An oropharyngeal swab and a capillary blood sample were taken from every child at each examination time point. The swab was used to determine if the child was acutely infected with SARS-CoV-2. Capillary blood was tested for SARS-CoV-2-IgG antibodies using a rapid test (dichotomous outcome) and a dried blood spot test (quantitative outcome). If either the rapid test or the dried blood spot (Euroimmun® ELISA) test resulted in a positive outcome for IgG antibodies, the child’s venous blood was drawn additionally for a more differential screening (n = 21) (S1 Fig). Parents and teachers/caregivers were subjected to the rapid test for IgG antibodies with capillary blood. If the result was positive, the blood was further examined with the dried blood spot test (S2 and S3 Figs).

At each examination, every participant received a questionnaire. The questionnaire for children and their parents contained questions about COVID-19 symptoms, social contacts, travelling abroad, hygienic behavior, rheumatologic diseases, and medication. In addition, parents were asked about their work situation. Parents were left to decide whether they completed the questionnaire for their child or children, with their child or children, or had the child or children complete it on his/ her or their own. These options were also recorded in each questionnaire. The questionnaires and consent forms were written in the most child-friendly language possible. Teachers and educators received the same questionnaire as parents. Each facility was given a questionnaire to assess what measures they took to reduce the transmission of SARS-CoV-2.

The data collection procedures were performed in accordance with the Declaration of Helsinki. Each participant gave written informed consent to participate in the study. The study was approved by the Ethics Committee of the Ludwig-Maximilians-Universität München (Number of the ethics vote: 20–0419).

### Laboratory analysis

Oropharyngeal Swabs (Sigma-Transwab®) were collected and tested on the same day by transcription mediated amplification (TMA, Hologic: Aptima SARS-CoV-2 with an analytical sensitivity of 0.01 TCID_50_/ml).

The applied rapid SARS-CoV-2-antibodytest is a lateral flow immunoassay (nal von minden, NADAL®) showing qualitative lines for IgG and IgM. We focused on the presence or absence of IgG only, ignoring possible IgM-lines. According to the manufacturer, diagnostic sensitivity is 85.2% for IgG 15 to 68 days after infection with SARS-CoV-2, and 60.6% within the time slot of 0 to 14 days. Diagnostic specificity is stated with 99.2% for the combination of IgM and IgG. This test detects antibodies to spike proteins.

All children donated a few additional capillary blood drops taken from their finger pads, which were soaked on a filter paper (Euroimmun®). The filter paper was stored in plastic bags for up to 14 days at 2–8°C and then stamped out with a Euroimmun® cutter to achieve exact amounts of dried blood spots. These dried blood spots were diluted and then measured quantitatively with the Euroimmun® Anti-SARS-CoV-2-QuantiVac-ELISA (enzyme-linked immunosorbent assay) (IgG). The ELISA reacts only to antibodies against spike protein and ignores antibodies to the nucleocapsid protein. The accordance of dried blood spots results to venous blood results is specified as 100% by Euroimmun®. Diagnostic sensitivity is determined to be 90.3% at least 10 days after infection and 93.2% for more than 20 days after infection with SARS-CoV-2. Specificity is reported to be 99.8%. Thus, two different antibody-screening tests were our standard for the detection of a past SARS-CoV-2 infection in children.

If either the rapid antibody test or the ELISA was positive for SARS-CoV-2 IgG in children, a venous blood sample was taken from the child and tested with an additional CoV-2-antibody-ECLIA (Roche®: Elecsys Anti-SARS-CoV-2 S) the following day as well as a Coronavirus Line Blot (Mikrogen®). The Roche® Anti-SARS-CoV-2-ECLIA detects IgG antibodies to the spike protein. It shows a sensitivity of 98.8% 14 or more days after infection and a clinical specificity of 99.9% according to the manufacturer.

The Mikrogen® *recom*Line SARS-CoV-2-IgG/ CE-IVD allows the differentiation of antibodies against seasonal coronaviruses (NP 229E, NP NL63, NP OC43 and NP HKU1) as well as the following proteins of SARS-CoV-2: NP SARS-2, RBD-SARS-2 and S1-SARS-2. Diagnostic sensitivity is stated as 85.7% for less than 12 days after infection, 95.2% for 12 to 23 days after infection and 100% if the infection occurred more than 23 days after diagnostics. This test has a very high diagnostic specificity according to the manufacturer with 97.9% for potentially cross-reactive samples and 99.7% in blood-donors.

Parents and teachers or caregivers were only tested with the rapid lateral-flow test. If their result was positive for IgG antibodies, the capillary blood of these participants was again examined via dried blood spots for SARS-CoV-2 antibodies using the Euroimmun® ELISA (see above).

### Statistical analysis

Descriptive analysis included the participation rate of schools and preschools. Medians and interquartile ranges (IQRs) were calculated. The proportion of positive tests (IgG) was determined. IgG antibodies measured by Euroimmun® ELISA were assessed in RE/ml, and then converted to Binding Antibody Units (BAU)/ml. BAU/mlSARS-CoV-2-IgG antibodies were presented stratified by positive/negative result for time-point comparisons and questionnaire data. When measuring IgG antibodies in the Euroimmun® ELISA, the lowest level of quantification is 2.4 BAU/ml. The highest measurable value was given as >288 BAU/ml. This value was set to 288.1 BAU/ml for all calculations. The percentage of not available values (missings/ NAs) is reported. NA values are excluded from the calculation of all statistical measures (arithmetic mean, median, percentage). For subgroup analyses, the size of the group is always reported in absolute numbers. Groups were first tested globally for differences using the Friedman test in the case of more than two groups. Pearson’s Chi-squared test, Fisher’s exact test, or Wilcoxon rank sum test were used when computing post-hoc tests or when testing two groups against each other. A statistically significant difference was assumed at a level of p<0.05. When making group-comparisons in symptomatic versus asymptomatic children, we divided symptoms into two groups: Symptoms since the start of the pandemic (fever, dry cough and loss of taste and/ or smell) and symptoms four weeks before baseline examination (sore throat, conjunctivitis, diarrhea, cold and extreme tiredness). Symptoms were grouped by time of onset (symptoms since the start of the pandemic and symptoms since 4 weeks before baseline examination). If the child ticked one symptom in either group of the groups, the corresponding variable was set as “Yes”. The R Studio ©, version 4.1.3, was used for statistical analyses.

## Results

A total of 365 children and 331 parents as well as 90 educators or teachers were included in the study. Almost 55% of the children were male and most of the children attended secondary school (40.5%). Of the participating children, 56.0% were in the age group 3–10 years, and 44.0% in the age-group 11–16 years. Mostly mothers (71.9%) and female educators and teachers (86.7%) took part in the study. The median age of the parents was 43.0 years (IQR 39.0; 47.0) and that of the teachers and educators 44.5 years (IQR 36.0; 53.0) (Table 1). The median interval between the measurement time points was 2 months between both baseline and the first follow-up (FUP1) and between FUP1 and the second follow-up (FUP2). At baseline, 7 (1.9%) children, 20 (6.0%) parents and 5 (5.6%) educators/ teachers reported having a positive PCR-test for SARS-CoV-2 in the past (Table 1).

**Table 1:** Descriptive statistics at baseline of the Augsburg Plus Cohort.

Variable	Children n = 365	Parents n = 331	Teachers or educators n = 90
Age in years: median (range) [IQR]	10.0 (3.0–16.0) [7.0–13.0]	43.0 (28.0–66.0) [39.0–47.0]	44.5 (18.0–62.0) [36.0–52.8]
Age group: 3–10 years (%)	206 (56.4)		
Age group: 11–16 years (%)	159 (43.6)		
Sex: male (%)	200 (54.8)	93 (28.1)	12 (13.3)
Sex: female (%)	165 (45.2)	238 (71.9)	78 (86.7)
Preschool (%)	64 (17.5)		37 (41.1)
Elementary school (%)	100 (27.4)		28 (31.1)
Secondary school (%)	148 (40.6)		13 (14.4)
Mixed school-type (%)	53 (14.5)		12 (13.3)
Previous SARS-CoV-2 infection*(%)	7 (1.9)	20 (6.0)	5 (5.6)

*Confirmed by PCR-testing.

**Fig 1:**
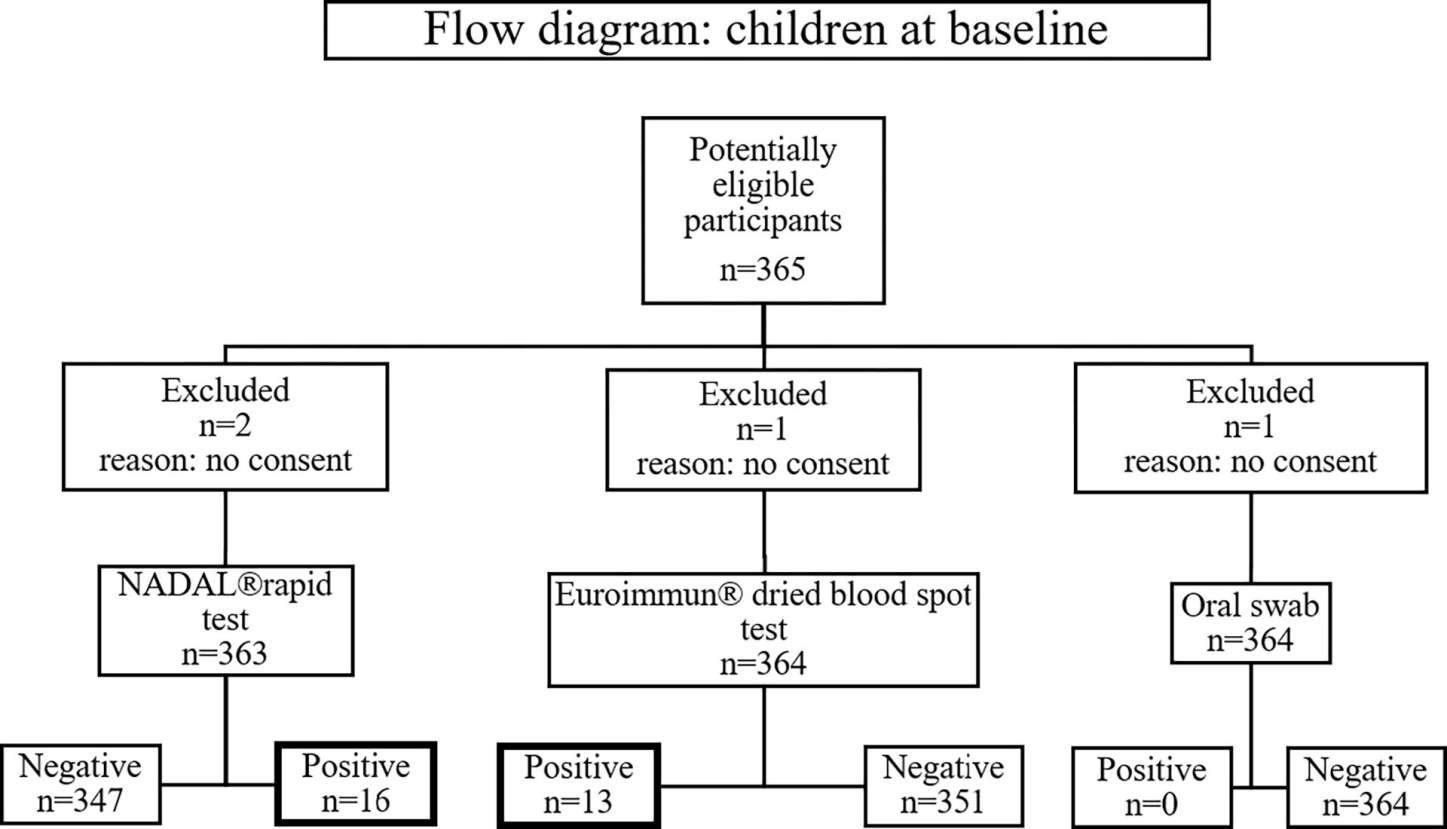
Flow diagram: Test-algorithm with number of tested children at baseline.

**Fig 2:**
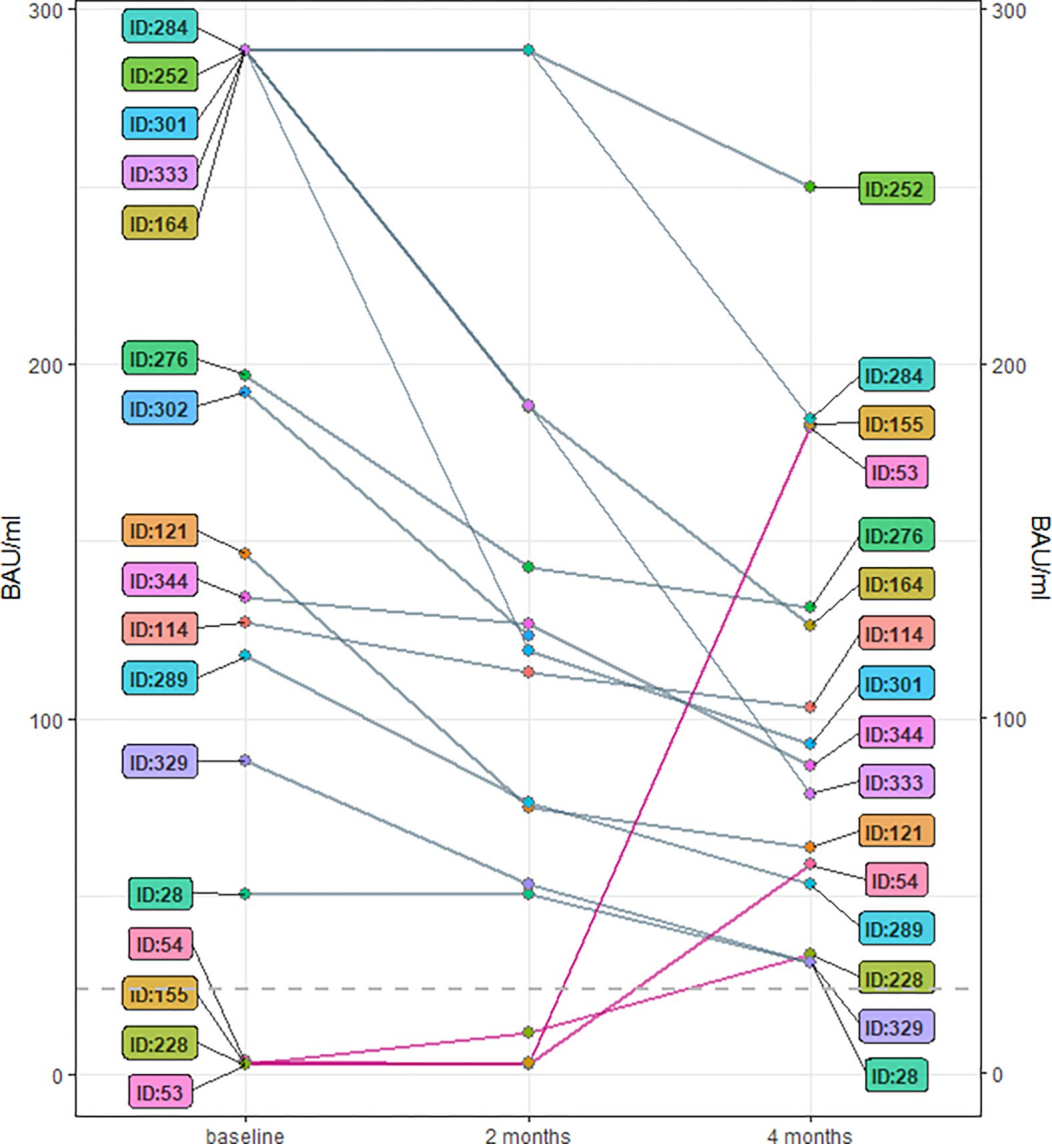
Before-and-after-plot: All children who tested positive for IgG-antibodies at least once during the study period. Every child is represented as an ID-number in the plot (n = 17 at baseline, and n = 16 at 4 months after baseline). The child with ID 302 was only tested at baseline and two months after baseline, so there was no third IgG-antibody value available. The grey dashed line at y = 24.0 BAU/ml represents the threshold for categorization into positive and negative results (a test result is IgG-positive at a level of ≥24.0 BAU/ml). All seroconverted children are indicated by a red line.

**Fig 3:**
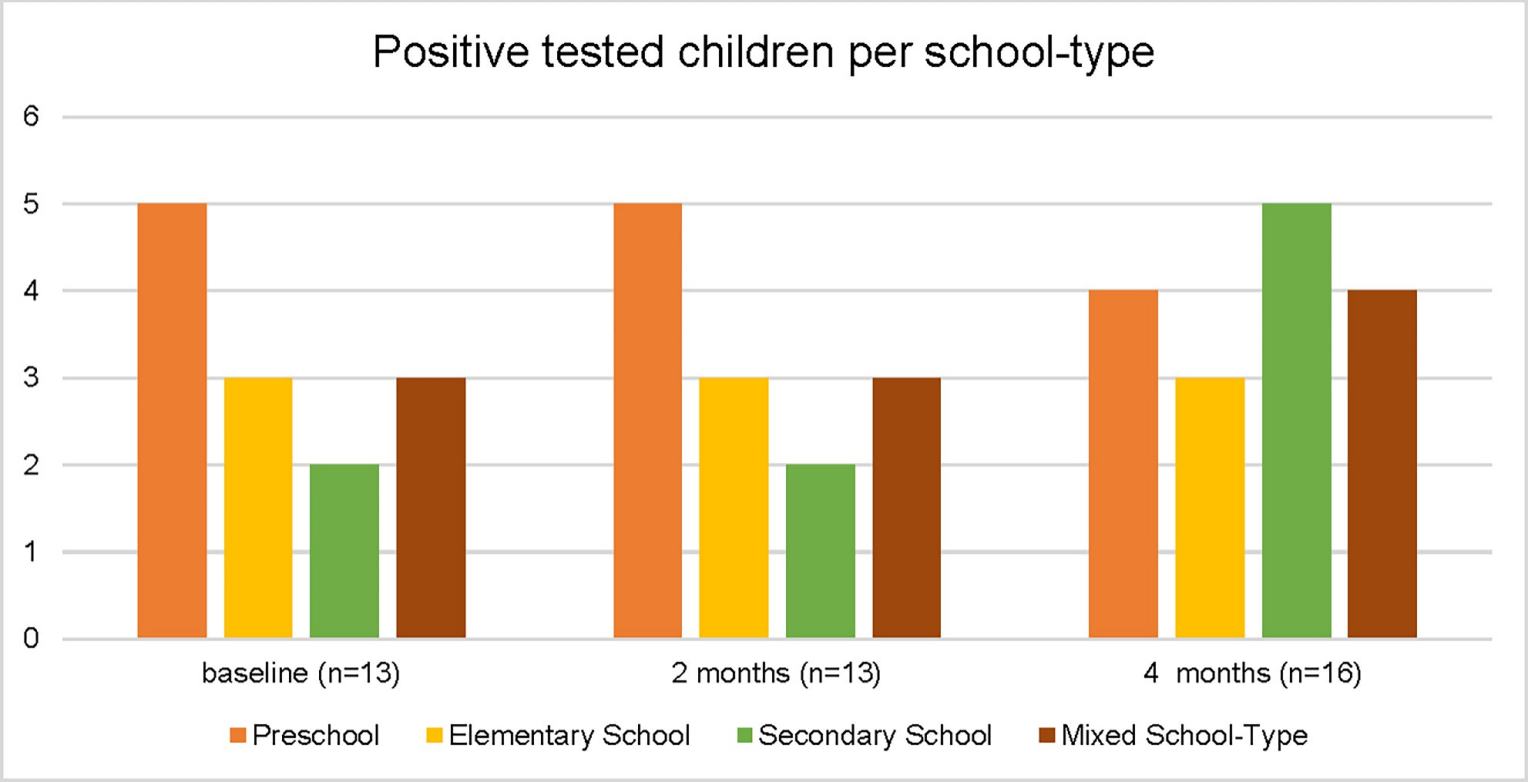
Children who tested positive per school-type (absolute numbers).

During the study period, no study participant tested positive for SARS-CoV-2 (n = 364 at baseline, Fig 1). At baseline, among 364 children tested, 13 (3.6%) children were positive for SARS-CoV-2-IgG antibodies in the Euroimmun® ELISA (Table 2). Of these 13 children, only 7 (1.9%) reported having a previous SARS-CoV-2 infection, verified by PCR-testing (Tables 1 and S3). We confirmed all seven known SARS-CoV-2 infections by measuring positive antibodies to spike protein 1 (SP1) (Roche® Anti-SARS-CoV-2-ECLIA) (S1 Table, child ID 329, 102, 284, 289, 302, 301, 252; S3 Table). Two children (ID 121 and 164), who did not report a PCR-confirmed SARS-CoV-2 infection in the past but were positive in the Euroimmun® ELISA and Roche® ECLIA test, reported symptoms (fever in December 2020 (ID 121) and fever with dry cough in February 2021 (ID 164)). After 2 months of follow-up, the same 13 children described at baseline tested positive for SARS-CoV-2 antibodies (Fig 2). Of those, most children attended preschool (Fig 3). In another follow-up about 4 months after baseline, 16 children tested positive for SARS-CoV-2 antibodies. Out of the 13 children who tested positive at baseline, one child could not be tested a third time (Fig 2). At this time, we detected four seroconversions (Fig 2) one of which, however, could not be verified as a true SARS-CoV-2-infection by Roche® ECLIA or Mikrogen® *recom*Line blot (S1 Table). Of those four, three occurred in children at secondary schools and one case at a mixed-school (age 8). Most children who tested positive for IgG antibodies 4 months after baseline were attending secondary school (Fig 3).

**Table 2:** IgG antibodies in parents and educators/ teachers at baseline.

Subgroup	NADAL® Rapid-Test	Subgroup	Euroimmun® ELISA IgG antibodies
	IgG-positive (%)[95% CI]		≥24 BAU/ml^a^ (%)[95% CI]
Children (n = 363)	**16 (4.4%) [2.6–7.2]**	Children (n = 364)	**13 (3.6%) [2.0–6.2]**
Parents (n = 297)	**17 (5.7%) [3.5–9.2]**	Parents (n = 16)	**13 (81.3%) [53.7–95.0]**
Educators/ Teacher (n = 81)	**4 (4.9%) [2.6–12.8]**	Educators/ Teacher (n = 4)	**3 (75.0%) [21.9–98.7]**

^**a**^The abbreviation “BAU” stands for Binding Antibody Units.

To get a proper look at the antibodies of the adults in the Augsburg-Plus cohort, all vaccinated participants were removed from the analyses. However, this also excluded 1 parent who had been infected at some time prior to the examinations. The exclusion of vaccinated adults reduces the sample of tested parents to n = 299 and the sample of teachers/ adults to n = 81 (Table 2).

13 parents (4.4%) tested positive for IgG antibodies at the baseline visit (Table 2). Only 12 of the IgG-positive parents in the NADAL®-rapid-test, and 9 of the IgG-positives parents in the Euroimmun® ELISA reported a known passed SARS-CoV-2 infection confirmed by PCR. The mean (standard deviation) duration between SARS-CoV-2 infection and baseline IgG in parents was 141.3 (123.2) and 160.7 (137.7) days for IgG-positives in the NADAL®-rapid-test and the IgG-positives in the Euroimmun® ELISA respectively. At the same time, of 5 reported SARS-CoV-2 infections in educators/ teachers we could confirm only 3 cases with positive IgG antibodies (Table 2). The mean duration between SARS-CoV-2 infection and baseline IgG in this group was 117.2 (52.8) and 112.0 (63.4) days for IgG-positives in the NADAL®-rapid-test and in the Euroimmun® ELISA, respectively. We detected one case of seroconversion in parents 4 months after baseline, but none in teachers/educators. The child of the seroconverted parent was also tested positive in the follow-up (S1 Table: TN-ID 53). In the questionnaire, the dates of the positive PCR-test-results were given, accordingly to which the parent was infected with SARS-CoV-2 first, the child thereafter.

**Fig 4:**
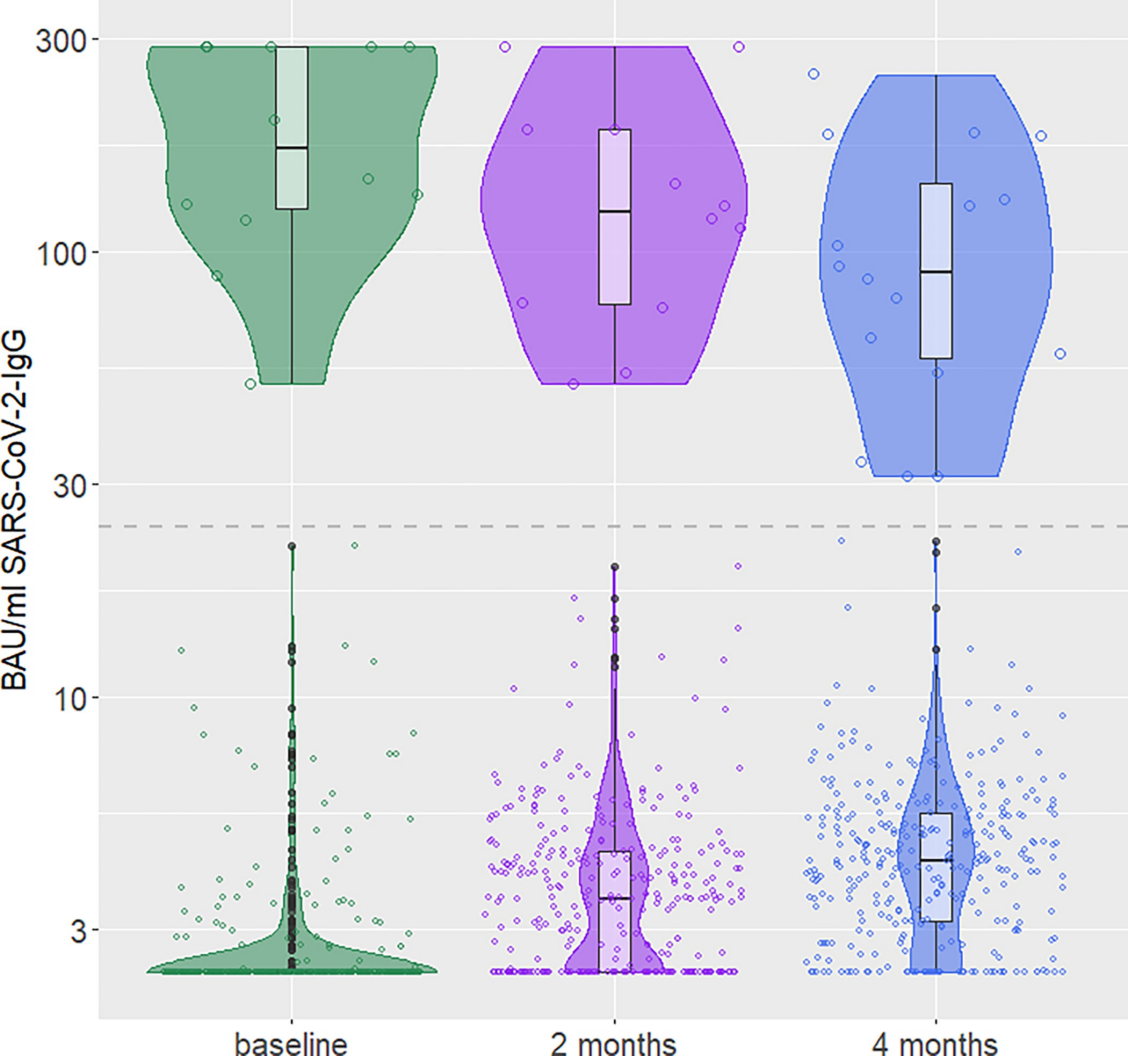
Violinplot SARS-CoV-2-IgG antibodies in BAU/ml over 4 months (dried blood spot: Euroimmun® ELISA) (n = 332). The Figure comes log10-scaled on the y-axis. Each violin presents one examination time point, stratified for positive and negative IgG antibody results. The grey dashed line at y = 24.0 BAU/ml indicates the threshold between IgG-positive and IgG-negative results (a test result is IgG-positive at a level of ≥24.0 BAU/ml, and negative at a level of <24.0 BAU/ml).

Fig 4 demonstrates the SARS-CoV-2-IgG antibody course (threshold = 24.0 BAU/ml) over the study period for all children with 3 testing time-points and no vaccination for SARS-CoV-2 (n = 332). Children with less than 3 testing time-points (Euroimmun® ELISA) are excluded from the figure. The number of children for each violin and median IgG antibody values (IQR) are presented in Table 3. When excluding all IgG-negative children and comparing the groups (baseline, 2-months, 4-months) by antibody values, there is a significant decrease in IgG antibodies between baseline and 4 months after baseline (p<0.05) (Table 4).

**Table 3:** Number of children in each violin of Fig 4, with median IgG antibody values (IQR) for each violin.

	Group	Median IgG in BAU/ml	IQR(0.25; 0.75) IgG in BAU/ml
Negatives	Baseline (n = 351)	2.4	2.4; 2.4
	2 months (n = 341)	3.5	2.4; 4.5
	4 months (n = 319)	4.3	3.1; 5.5
Positives	Baseline (n = 13)	192.0	127.2; 288.2
	2 months (n = 13)	123.6	76.6; 187.7
	4 months (n = 16)	89.9	57.4; 144.2

**Table 4:** Differences in IgG antibody levels (Euroimmun® ELISA) for IgG-positive children between time points.

Groups (n = 12)	p-value
Baseline-2 months	0.102
Baseline-4 months	0.004
2 months-4 months	0.108

**Fig 5:**
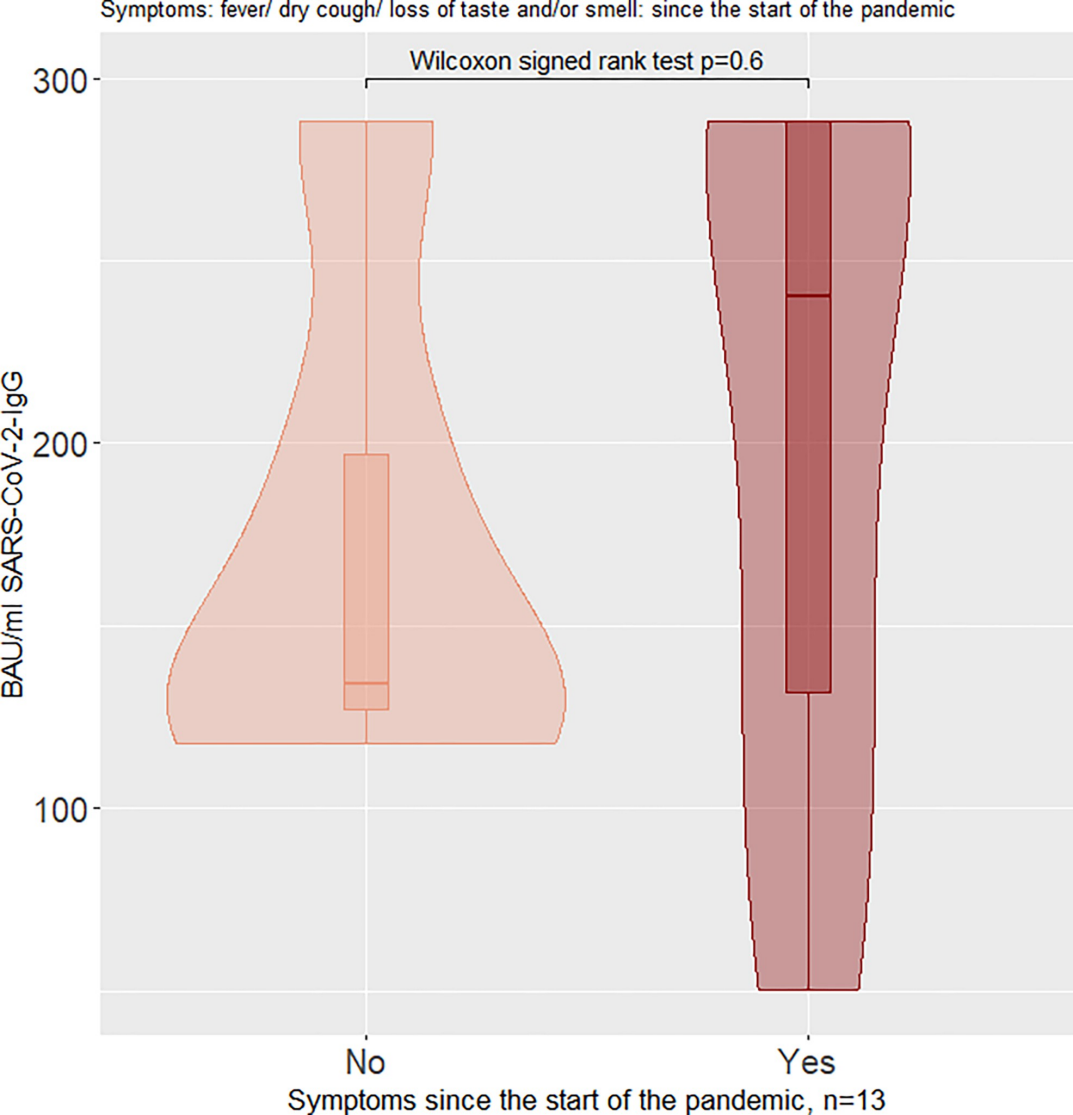
Violin plot of SARS-CoV-2-IgG antibodies in BAU/ml at baseline (Euroimmun® ELISA), stratified for symptomatic and asymptomatic children: Symptoms since the start of the pandemic.

**Fig 6:**
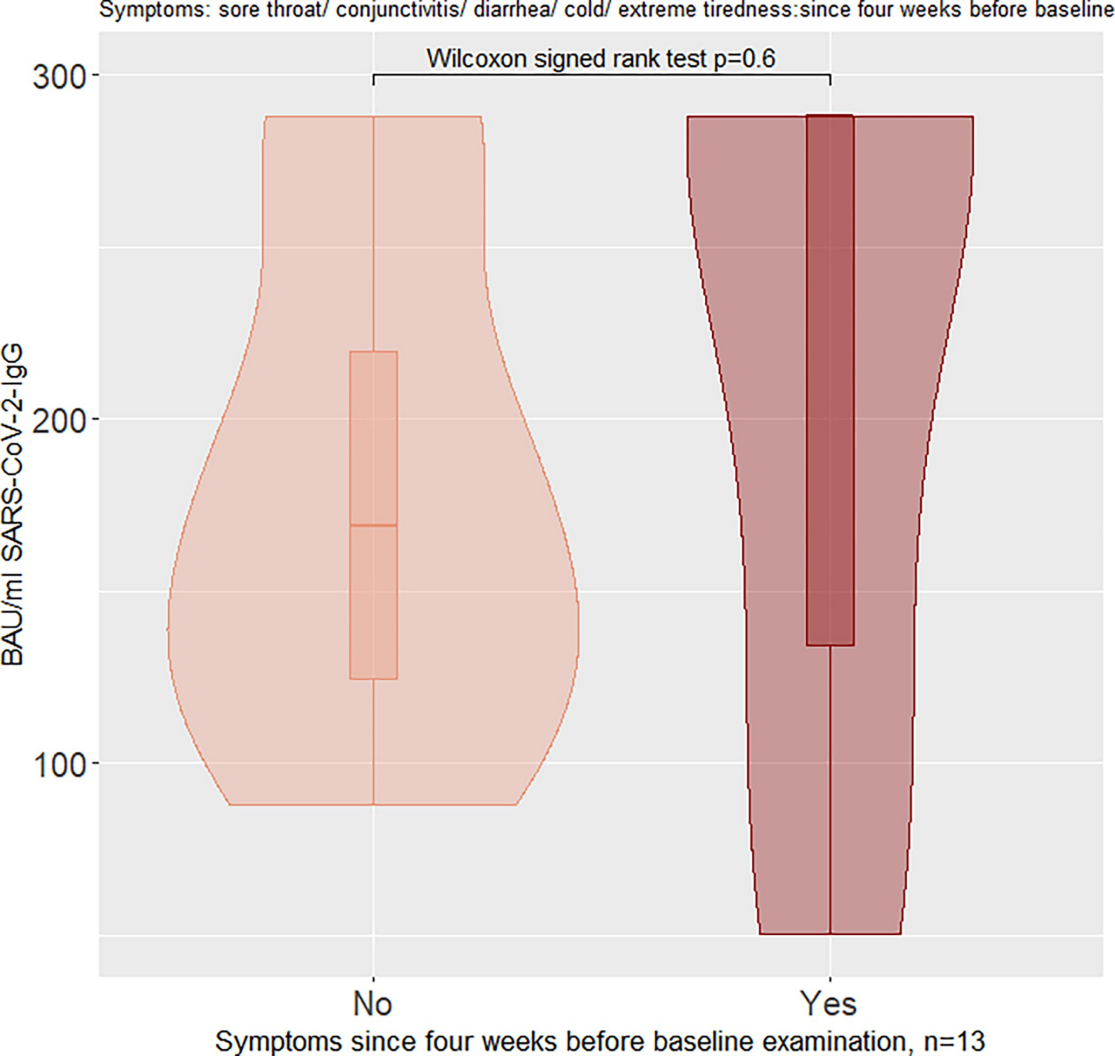
Violin plot of SARS-CoV-2-IgG antibodies in BAU/ml at baseline (Euroimmun® ELISA), stratified for symptomatic and asymptomatic children: Symptoms four weeks before baseline examination.

When looking at IgG-positive children at baseline, children with symptoms had higher median IgG levels (BAU/ml), but the difference between symptomatic and asymptomatic children was not significant (Figs 5 and 6). The number of children per subgroup and median values (IQR) for Figs 5 and 6 are presented in Table 5.

**Table 5:** Number of children in each violin in Figs 5 and 6, with median IgG antibody values and IQRs for each violin.

	Group	Median IgG in BAU/ml	IQR(0.25; 0.75) IgG in BAU/ml
Symptoms since the start of the pandemic	Yes (n = 8)	240.1	131.8; 288.2
	No (n = 5)	134.2	127.2; 196.8
Symptoms 4 weeks before baseline examination	Yes (n = 8)	288.2	134.2; 288.2
	No (n = 5)	169.2	124.8; 219.7

When stratifying the children by IgG antibody status (positive/negative), we found that significantly more IgG-positive children also had an IgG-positive parent (p<0.001). In addition, significantly more IgG-positive children also had a positive PCR test (p<0.001), and/or one parent had a positive PCR test (p<0.001) before study entry. All IgG-positive children had contact with a person with COVID-19 at some point (p<0.001). Looking at the hygiene behavior of the children, significantly more IgG-negative children are in the group of those who wash or disinfect their hands more frequently since the start of the pandemic (p = 0.037). There was no significant difference in IgG antibody outcomes between facility types (S2 Table). Nevertheless, the facilities with IgG positive children are presented below.

Table 6 presents an overview of all IgG-positive children at baseline, stratified by the facility (listed are school type and acronym of institution name) the children attended. Most IgG positive children also had an IgG-positive parent. If IgG-positive teachers/educators were present at an institution, at least one child was also IgG-positive in each case (Table 6).

**Table 6:** Facilities which the IgG-positive children attended, with associated IgG-positive parents and number of IgG-positive teachers/educators working in each facility.

	Children	Parent	Teacher/ Educators	Context
Elementary and secondary school a. E.	**3**	**3**	0	Children each with an infected parent
Elementary School S.	**1**	**2**	0	Child is not related to the infected parents
Elementary School W.	**1**	**1**	0	Child with infected parent
High school St. A.	**1**	**3**	0	Child with infected parent plus two unrelated, infected parents
Preschool D. W.	2	0	0	Children without an infected parent
Preschool St. G. S.	2	0	0	Children without an infected parent
Elementary School P. G.	**1**	**1**	**1**	Child with infected parent
Secondary School Z.	1	0	0	Child without an infected parent
Preschool T. S.	**1**	0	**2**	Child without an infected parent

The S1 Table shows all children (n = 21) who tested positive for IgG antibodies using the NADAL® rapid test or the Euroimmun® ELISA using dried blood spots at least at one examination during the study period. Two children never tested positive on the rapid test, but tested positive at least once on the Euroimmun® ELISA (ID 28 and 228). Each child listed in the S1 Table had venous blood drawn a second time, except for the children with the IDs 284, 329, 333, and 164 because they had not consented to venous blood sampling. The weakly positive antibody-detection in the Euroimmun® ELISA of ID 228 was probably due to a cross-reactivity to seasonal coronaviruses NL63 and/or 229E. We were able to confirm a previous SARS-CoV-2 infection in 15 of the children who consented for venous blood sampling using Roche® ECLIA (antibodies to the SP1 were detected in these children). We were able to line-blot (Mikrogen®) the venous blood samples of 13 children and could confirm a past SARS-CoV-2 infection by measuring antibodies to the receptor binding domain (RBD), spike protein and/or nucleocapsid protein (NCP). We found agreement between all samples in which a SARS-CoV-2-infection was identified via the detection of NCP-, RBD- and/or SP1-IgG in the Mikrogen® *recom*Line blot and those in which an infection was identified via the detection of IgG to SP1 in the Roche® ECLIA. However, one sample with a weak IgG detection in both Roche® ECLIA and *recom*Line blot was below the cut-off in the Euroimmun® ELISA using dried blood spot.

Of the 16 children who tested positive for antibodies to SP1 during the entire study period, only nine reported having a previous SARS-CoV-2 infection (S1 Table). In a recheck of available plasma, all samples with positive SP1 were also positive in the NADAL rapid test for IgG antibodies. For 11 of the 16 children listed in S1 Table, at least one parent was infected with SARS-CoV-2 and experienced symptoms. One child (ID 28) tested weakly positive for SARS-CoV-2-IgGs in the ELISA at every time point. Therefore, we invited the child for venous blood sampling. However, at the time of the blood sampling, the child had already been vaccinated once. Thus, the RBD- and SP1- antibodies were likely mainly due to the vaccination. Antibodies to NCP were missing in this child, as well as antibodies to seasonal coronaviruses possibly due to NCP´s shorter time of persistence, which is described by the manufacturer. However, the non-detectability of NCP could also be due to a false-negative result in the screening assays. The children with ID 53 and 54 (siblings) reported having been infected with SARS-CoV-2 in April 2021 (one month before blood sampling) and still reported a sore throat one month after the infection. ID 53 still showed IgA-antibodies to SARS-CoV-2-spike-protein. Two cross-reactivities in the NADAL® rapid test with seasonal coronavirus NL63-antibodies occurred in the children with ID 132 and 228. Both showed no reaction in the Roche® ECLIA, though one of them showed a small reaction (33.6 BAU/ml) in the dried blood spot ELISA.

## Discussion

In the present study, SARS-CoV-2 seroprevalence of 3.6% (n = 13) was measured at baseline using Euroimmun® dried blood spots. The adults in our cohort had a comparable or slightly higher prevalence as observed in the children: We measured an IgG seroprevalence of 4.3% (n = 13) and 3.7% (n = 3) for parents and educators/teachers at baseline, respectively.

Twelve of the IgG-positive children could be confirmed by the Roche® Anti-SARS-CoV-2-ECLIA using venous blood samples (except for ID 333, who refused venous blood sampling). However, at baseline only six (50.0%) of the 12 children with confirmed SARS-CoV-2 infections reported having been ill with the disease–suggesting an underdiagnosis of SARS-CoV-2 infections in children during the Augsburg-Plus study period. Our finding of a 50.0% underdiagnosis of SARS-CoV-2 infection in children is in accordance with the results of two further investigations [10,11], although the extent of underdiagnosis that was reported in some other studies varies [12–16]. No child in our cohort tested positive for SARS-CoV-2 during the period of data collection. Symptomatic children (at the time of the planned visit) were asked to arrange a new appointment. In contrast, the “Münchner Virenwächter” [17] identified two SARS-CoV-2 -positive children in 2020 when testing children from 5 primary schools and 5 preschools in Munich, not excluding children with symptoms. The reason for the discrepancy in the number of cases identified during the study may be due to the exclusion of symptomatic children in our study.

To date, few studies have measured SARS-CoV-2-specific IgG antibodies in children in order to determine whether a child has previously experienced a symptomatic or asymptomatic infection with SARS-CoV-2 [9,12–14,18–24]. Studies on this issue are rather important, as it remains unclear what role children play in the transmission of (severe) SARS-CoV-2 infections and what the prevalence of SARS-CoV-2 infection is in children without symptoms [7].

It may be assumed that immunity following a SARS-CoV-2 infection lasts approximately as long as with SARS-CoV, which is up to 24 months after infection [18]. The formation of IgG antibodies occurs at about 14–20 days post onset of symptoms [18].

SARS-CoV-2 seroprevalence in our study was approximately 3.5%, which is higher than what was measured in a Swiss (regarding the group of children aged 5–9 years [21]), and two German studies [22,23] (Table 7). However, one Italian, one German, and two Swiss studies measured prevalences ranging from 5.6 to 9.6% [13,19–21]. On the other hand, Stringhini et al. [21] measured a higher prevalence (age group: 10–19 years) (Table 7). The higher detection of SARS-CoV-2 antibodies in a study named “Fr1da” (“Typ-1-Diabetes: Früh erkennen–Früh gut behandeln”) [20] might be due to different regions in Bavaria, the study was conducted in a different season, or other methods of testing. Furthermore, it was not mentioned whether cross-reactivity to seasonal coronavirus NL63 was evaluated [20].

**Table 7:** Overview: Studies measuring seroprevalence in children.

Author	Location	Period of Data collection	Age (years) range	N	Seroprevalence (%)	
Pagani et al. [19]	Castiglione d’Adda, Italy	05/18/2020-06/07/2020	0–19	61	8.2%	
Hippich et al. [20]	Bavaria, Germany	10/2020-02/2021	1–10	26,903	pre-school: 5.6% (95% CI, 4.7%– 6.7%)	
					school children: 8.4% (95% CI, 6.4%–10.9%)	
Posfay-Barbe et al.[13]	Geneva, Switzerland	04/01/2020-04/30/2020	3.6–13.3	208	8.7%	
Stringhini et al. [21]	Geneva, Switzerland	04/06/2020-05/09/2020	5–9	123	0.8%	7.3%
			10–19	332	9.6%	
Tonshoff et al. [22]	Baden-Württemberg, Germany	04/22/2020-05/15/2020	0–10	2482	0.6% [95% CI, 0.3–1.0%]	
Hommes et al. [23]	Berlin, Germany	07/11/2020-07/19/2020	primary school: 8–13	382	1.8%	
			secondary school: 13–18			
Rostami et al. [24]	23 WHO regions/ countries (systematic Review)	studies published up to August 2020	<19 years	18,333	2.3% [95% CI 1.0–3.6]	

Rostami et al. conducted a systematic review of seroprevalence in the general population with studies published up to August 2020 worldwide. For children/adolescents younger than 19 years, a seroprevalence of 2.3% (95% CI 1.0–3.6) was calculated when reviewing all data sets [24].

Hommes et al. conducted a cohort study in Berlin, which is comparable to our study. The data collection took place in June 2020. In this study, children and teachers from various primary and secondary schools were also recruited. They identified only one positive PCR test over the course of the study period in a 16-year-old girl among a total sample of 382 tested participants [23]. However, the measured IgG seroprevalence of 1.8% (n = 7) was lower than in the present study. This indicates that the SARS-CoV-2 incidences of Berlin and Augsburg at the respective time of data collection differed strongly. The Berlin data were collected from 07/11/2020-07/19/2020 [23]. On the last day of data collection, a 7-day incidence of 77.6/100,000 was measured in Berlin [25]. Augsburg data at the time of the last follow-up was collected between April and August 2021. On 04/07/2021, a 7-day incidence peak of 276.5/100,000 was recorded for the city of Augsburg and maintained >100/100,000 until mid of May 2021 [26]. When looking at the different periods of data collection, in addition to the incidence of SARS-CoV-2, the variants of concern (VOC) of SARS-CoV-2 must also be examined. During our data collection, the VOC delta of SARS-CoV-2 emerged, a VOC that transmits more rapidly than the predecessor variants. As of the second week of May 2021, the proportion of VOC delta of the genome sequences in Germany was estimated to be 40% [27]. Furthermore, the more adults are vaccinated, the more children may have a greater impact on the spread of VOC [28,29].

In our study, six of a total of twelve confirmed seropositive children (50.0%) at baseline did not know that they had undergone SARS-CoV-2 infection. The finding of an underdiagnosis of SARS-CoV-2 infection in children is in accordance with two other investigations [10,11] although the extent of underdiagnosis varied between studies [12–14].

IgG antibodies decreased significantly over time but persisted in 100% over a period of 4 months in our study. This confirms the result of Renk et al., who still measured IgG antibodies in 96.2% of their cohort including 548 children aged 6–13 years 11–12 months after baseline examination [9]. Considering that the risk of reinfection since the outbreak of the VOC omicron is higher than for the VOCs beta and delta, the question arises of how long IgG antibodies produced by SARS-CoV-2 infection can provide protection [30].

Altogether, three seroconversions occurred during the 4-months follow-up, two of which occurred in children attending secondary school. In the parents of the children, we detected one case of seroconversion, and none in educators/teachers. These cases must be seen in the context of the incidence of infection in the population and the public measures taken to contain the pandemic. The comparatively few to no seroconversions among adults in the cohort may be due to the high vaccination-rate. Within 4 months after baseline, 66.1% of the parents, and 94.1% of educators/teachers had been vaccinated at least once. Fischer et al. measured an IgG seroprevalence of 0.9% within a cohort of more than 3000 adults from three German states, who donated blood between March 2020 and June 2020 [31], which is far below our measured prevalence one year later. On the contrary, a population-based study from northern Serbia reported a seroprevalence of 6.9% in 30–64 years old individuals at the end of June 2020 [32]. Seroprevalence of SARS-CoV-2 antibodies in adults varied in studies from <0.1% to >20.0% [21,33–37]. The examinations 4 months after baseline took place between April and the end of August 2021 for all participants. On 03/15/2021, measures to control the pandemic for elementary and secondary schools were eased for the city and county of Augsburg [38]. For example, from 05/19/2021 to the end of June 2021, school classes were divided with one part attending presence classes and the other working online (“Wechselunterricht”), given that a 7-day incidence of 100/100,000 was not exceeded. Beginning in July 2021, due to a 7-day incidence of <50/100,000 in the city and county of Augsburg, in-presence classes were allowed for all pupils and students until summer break [26]. Students wore face masks most of the time during classes, whereas children in preschool did not. Still latter group didn’t show any seroconversion during this period of time. In addition, children and adolescents who attend secondary school are at an age when they have the highest number of social contacts. Mossong et al. found the highest number of social contacts among children and adolescents aged 10–19 [39]. This, in turn, could have influenced the transmission rate of SARS-CoV-2. Nearly 60% of the children in our cohort reported meeting 1–2 friends, 1–2 times a week, but we measured no significant difference when comparing IgG-positives with–negatives.

We demonstrated the capability of antibody testing out of capillary blood in children and used two different systems, including an ELISA out of dried blood spots (Euroimmun®) by comparing these results to antibodies from venous blood samples. Furthermore, we detected that two of the children who screened positive by NADAL® rapid test were actually infected with the seasonal human coronavirus NL63 instead, and one additional child with a soft reaction in IgG had no proof of SARS-CoV-2-antibodies in any further assay.

### Limitations

The results of the present study could be biased by the fact that participation in the study was based on voluntary enrolment by parents. However, we tried to counteract this by restricting participation only to children from the participating facilities. Within the facilities, the facility managers repeatedly called for participation. Unfortunately, we could recruit comparatively fewer children from preschools than from other facilities for the study. We tried to compensate for this by working with more preschools. As the vaccination campaign for adults started while our study was conducted, we were somewhat limited in terms of determining seroprevalence. In addition, children who may have developed only a cellular immune response (T-cells) remained undetected in our study. Furthermore, the results always have to be considered in the context of the different phases of the pandemic, the differently implemented measures of outbreak control, the included study participants and the serologic test used for the antibody determination. The assays we used perform differently depending on how many days after symptom onset the test is done [40]. In addition, the measured prevalence values may be more or less reliable depending on the sample size [41–44].

### Strengths

One strength of the study is first and foremost that we used two different test systems for the screening for antibodies and were able to obtain a more accurate picture of actual SARS-CoV-2 cases by recomLine blotting than was initially indicated by ELISA or even rapid testing. This allowed us to sort out those antibody reactions that were due to cross-reactivity to seasonal coronaviruses (NP 229E, NP NL63, NP OC43 and NP HKU1).

Multiple study time points also allowed us to capture seroconversions. Our study took place during the third wave of the pandemic, i.e. at a more recent point in time than previous studies on seroprevalence. Thus, our results reflect current conditions on seroprevalence in 3-16-year-old children and complement previous studies on this topic.

## Conclusions

The combination of two antibody test systems, both out of capillary blood, proved to be a reasonable way to test children with minimal invasiveness combined with an acceptable sensitivity. Results of our study showed a seroprevalence of SARS-CoV-2 antibodies of 3.6% in children aged 3–16 years. However, it seems that SARS-CoV-2 infections in children were underdiagnosed during the time of data collection of the Augsburg Plus data. More data on SARS-CoV-2 seroprevalence in children will be useful, especially longitudinal data, which map the IgG antibody course over a longer time period. In Germany, 20.0% children of the 5–11 year old children of age are currently vaccinated [45]. For the 4.0 million people aged 0 to 4 years living in Germany, no licensed vaccine is available yet [45]. Thus, monitoring of this age group would be of great interest. To counteract the number of unreported cases of SARS-CoV-2, studies on antibody measurement are a good instrument to generate further evidence on the observed trends of the infection dynamic in children.

## Supporting information

S1 Fig Flow diagram: Blood sampling in children throughout the study. (TIFF)

S2 Fig Flow diagram: Test-algorithm for parents. (TIFF)

S3 Fig Flow diagram: Test-algorithm for teachers/ educators. (TIFF)

S1 Table Details of examination of the blood of children with positive IgG-Antibodies in the NADAL rapid test and/or Euroimmun-ELISA from dried blood spots. (PDF)

S2 Table Questionnaire data of the children stratified by Euroimmun® ELISA result (positive/ negative). (PDF)

S3 Table Overview with children who were positive in ELISA or ECLIA. (PDF)
