# Supplementary figures and images for: Longitudinal change in SARS-CoV-2 seroprevalence in 3-to 16-year-old children: The Augsburg Plus study
Source: PLoS One. 2022 Aug 11;17(8):e0272874. doi: 10.1371/journal.pone.0272874 (PMC9371315; doi:10.1371/journal.pone.0272874)

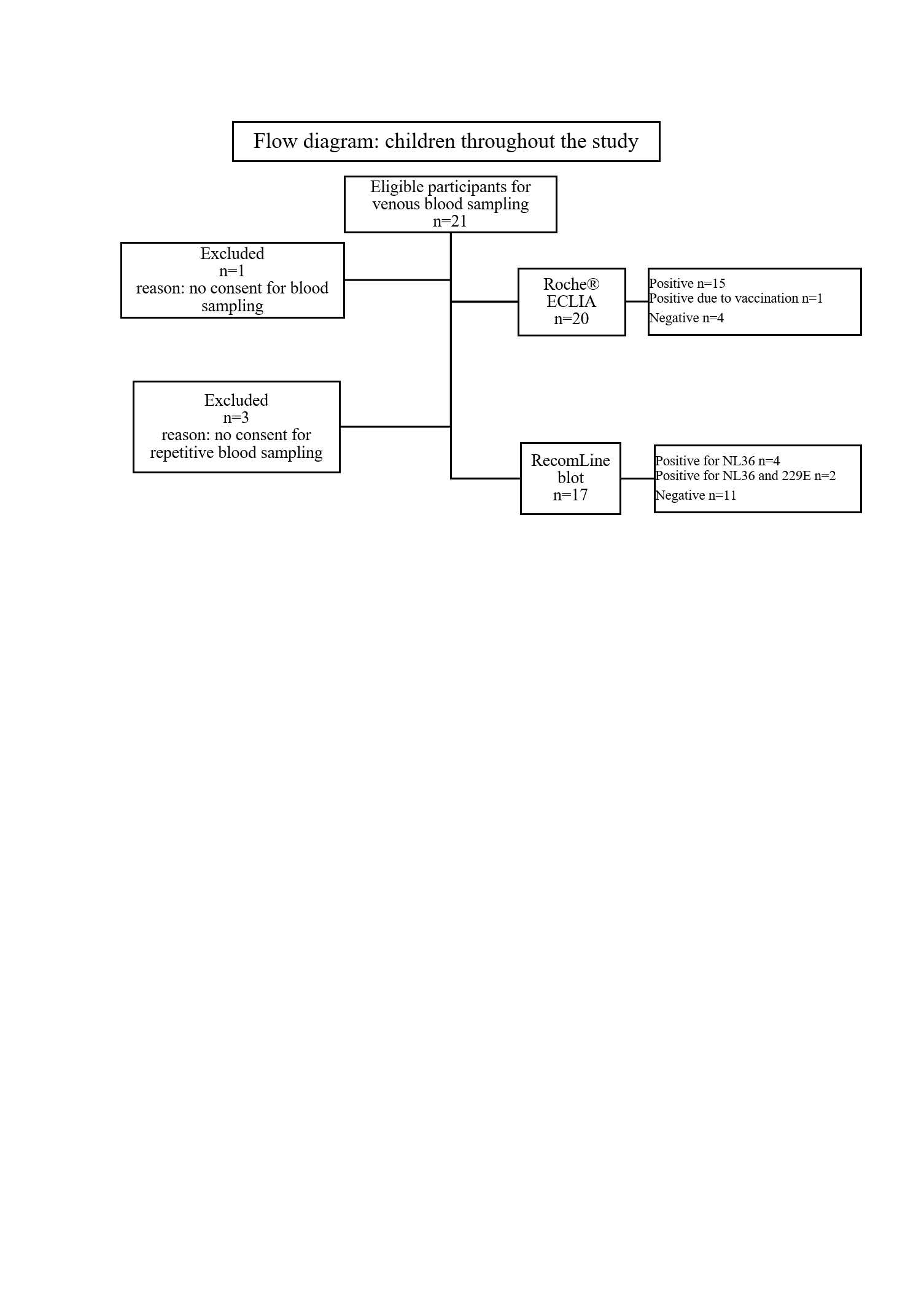

Supplement: S1 Fig — (TIFF) [file pone.0272874.s001.tiff]
