# Supplementary material for: Longitudinal change in SARS-CoV-2 seroprevalence in 3-to 16-year-old children: The Augsburg Plus study
Source: PLoS One. 2022 Aug 11;17(8):e0272874. doi: 10.1371/journal.pone.0272874 (PMC9371315; doi:10.1371/journal.pone.0272874)
Supplement: S1 Table — (PDF) [file pone.0272874.s004.pdf]

ID	Age	Gender	Time Point of the first positive rapid Test (NADAL®) (IgG)	Sample-collection at	Euroimmun® IgG BAU/ml	Sample-collection at	Roche® ECLIA: Spike Protein-IgG U/ml	IgG antibodies to seasonal Coronaviruses	SARS-IgG-Line-Blot			SARS-IgA-Line-Blot			NADAL®-Rapid-Test Control	Seroconversion in FUP2	Known SARS-CoV-2 infection
					Values <24 are interpreted as negative		Values <0,8 are interpreted as negative	Test for following Antibodies: 229E, NL63, OC43, HKU1	Nucleocapsid protein	Receptor binding domain	Spike Protein 1	Nucleocapsid protein	Receptor binding domain	Spike Protein 1	IgG		
132	10	female	Baseline	Baseline	< 12	3 Month after FUP2	< 0.4	NL63							X (weak)		no
**155**	**8**	**male**	**FUP2**	**FUP2**	**183.12**	**FUP2**	**116**	**NL63**	**X**	**X**	**X**				**X**	**X**	**no**
**102**	**11**	**male**	Baseline	**FUP1**	16.56	**FUP1**	**11.10**	**negative**	**X (weak)**								**yes**
287	5	male	Baseline	Baseline	< 12	Baseline	< 0.4	negative							X (weak)		no
**276**	**4**	**male**	Baseline	**Baseline**	**196.8**	**Baseline**	**307**	**negative**	**X**	**X**	**X**				**X**		**no**
**54**	**12**	**female**	**FUP2**	**FUP2**	**58.8**	**FUP2**	**9.60**	**negative**	**X**	**X**	**X**				**X**	**X**	**yes**
**301**	**13**	**male**	**Baseline**	**Baseline**	**> 288**	**Baseline**	**230**	**NL63**	**X**	**X**	**X**		**X**	**X**	**X**		**yes**
**289**	**11**	**male**	**Baseline**	**Baseline**	**117.6**	**Baseline**	**207**	**negative**	**X**	**X**	**X**				**X**		**yes**
**252**	**10**	**male**	**Baseline**	**Baseline**	**> 288**	**Baseline**	**814**	**229E, NL63**	**X**	**X**	**X**	**X**			**X**		**yes**
**53**	**15**	**female**	**FUP2**	**FUP2**	**182.16**	**FUP2**	**67.3**	**negative**	**X**	**X**	**X**			**X**	**X**	**X**	**yes**
**302**	**3**	**female**	**Baseline**	**Baseline**	**192**	**Baseline**	**378**	**negative**	**X**	**X**	**X**				**X**		**yes**
286	3	male	Baseline	Baseline	16.8	Baseline	<0.4	negative							X (weak)		no
**121**	**7**	**female**	**Baseline**	**Baseline**	**146.4**	**Baseline**	**201**	**NL63**	**X**	**X**	**X**			**X**	**X**		**no**
**114**	**8**	**male**	**Baseline**	**Baseline**	**127.2**	**Baseline**	**374**	**negative**	**X**	**X**	**X**				**X**		**no**
**344**	**4**	**female**	**Baseline**	**Baseline**	**134.16**	**Baseline**	**628**	**negative**	**X**	**X**	**X**			**X**	**X**		**no**
228	14	female	At each Time Point negative	FUP2	**33.6**	1 Month after FUP2	<0.4	229E (weak), NL63								X	no
28	15	male	At each Time Point negative	FUP2	**31.2**	3 Month after FUP2	**192***	negative		X*	X*		X*	X*	X*		no
**284**	**12**	**male**	**Baseline**	**Baseline**	**> 288**	**Baseline**	**252**	No Serum available									**yes**
**329**	**6**	**male**	**Baseline**	**FUP1**	**53.28**	**FUP1**	**221**										**yes**

**333**	**4**	**male**	**Baseline**	**Baseline**	**> 288**	No Serum available			no
**164**	**9**	**female**	**Baseline**	**Baseline**	**> 288**	**Baseline**	**430**	No Serum available	**no**
