# Supplementary material for: Longitudinal change in SARS-CoV-2 seroprevalence in 3-to 16-year-old children: The Augsburg Plus study
Source: PLoS One. 2022 Aug 11;17(8):e0272874. doi: 10.1371/journal.pone.0272874 (PMC9371315; doi:10.1371/journal.pone.0272874)
Supplement: S2 Table — (PDF) [file pone.0272874.s005.pdf]

Children stratified by Euroimmun ® ELISA test result	N	Positive, N = 13[1]	Negative, N = 351[1]	p-value[2]
**Sex**	364			0.3
Male		9 (69%)	191 (54%)	
Female		4 (31%)	160 (46%)	
**Age children**	364	8.0 (4.0, 11.0)	10.0 (7.0, 13.0)	0.11
**Vaccinated**	364			
No		13 (100%)	351 (100%)	
**Children who took a PCR-test in the past**	363			**0.047**
Yes		11 (85%)	199 (57%)	
No		2 (15%)	151 (43%)	
Missings		0	1	
**Result PCR-test in the past**	348			**<0.001**
Not applicable		2 (15%)	140 (42%)	
Positive		6 (46%)	2 (0.6%)	
Negative		5 (38%)	193 (58%)	
Missings		0	16	
**Had contact with a COVID-19 diseased person**	361			**<0.001**
Yes		12 (100%)	87 (25%)	
No		0 (0%)	262 (75%)	
Missings		1	2	
**Had fever since the start of the pandemic**	361			0.3
Yes		5 (42%)	101 (29%)	
No		7 (58%)	248 (71%)	
Missings		1	2	

Children stratified by Euroimmun ® ELISA test result	N	Positive, N = 13[1]	Negative, N = 351[1]	p-value[2]
**Had cough since the start of the pandemic**	361			0.3
Yes		4 (36%)	83 (24%)	
No		7 (64%)	267 (76%)	
Missings		2	1	
**Could not smell or taste anything since the start of the pandemic**	361			0.064
Yes		2 (15%)	10 (2.9%)	
No		11 (85%)	338 (97%)	
Missings		0	3	
**4 weeks before screening: had sore throat**	362			0.4
Yes		2 (15%)	33 (9.5%)	
No		11 (85%)	316 (91%)	
Missings		0	2	
**4 weeks before screening: had conjunctivitis**	359			>0.9
Yes		0 (0%)	3 (0.9%)	
No		13 (100%)	343 (99%)	
Missings		0	5	
**4 weeks before screening: had diarrhea**	363			0.6
Yes		2 (15%)	36 (10%)	
No		11 (85%)	314 (90%)	
Missings		0	1	
**4 weeks before screening: had cold**	360			>0.9
Yes		2 (15%)	60 (17%)	
No		11 (85%)	287 (83%)	
Missings		0	4	

Children stratified by Euroimmun ® ELISA test result	N	Positive, N = 13[1]	Negative, N = 351[1]	p-value[2]
**4 weeks before screening: had extreme tiredness**	357			0.5
Yes		1 (7.7%)	18 (5.2%)	
No		12 (92%)	326 (95%)	
Missings		0	7	
**Rheumatological disease**	362			
No		13 (100%)	349 (100%)	
Missings		0	2	
**Taking medication**	360			0.3
Yes		2 (15%)	25 (7.2%)	
No		11 (85%)	322 (93%)	
Missings		0	4	
**Been abroad since the beginning of the pandemic**	360			>0.9
Yes		7 (54%)	189 (54%)	
No		6 (46%)	158 (46%)	
Missings		0	4	
**Been in quarantine since the beginning of the current school year**	359			**<0.001**
Yes		11 (85%)	98 (28%)	
No		2 (15%)	248 (72%)	
Missings		0	5	
**Wash or disinfects hands more often**	358			**0.037**
Yes		8 (67%)	309 (89%)	
No		4 (33%)	37 (11%)	
Missings		1	5	

Children stratified by Euroimmun ® ELISA test result	N	Positive, N = 13[1]	Negative, N = 351[1]	p-value[2]
**Washing hands times per day**	33<br>1	15.5 (13.0, 18.5)	13.0 (10.0, 17.0)	0.2
Missings		3	30	
**Disinfect hands times per day**	29<br>5	7.0 (2.0, 8.0)	5.0 (2.0, 7.0)	0.5
Missings		5	64	
**Wears mask regularly**	36<br>0			0.058
Yes		8 (62%)	289 (83%)	
No		5 (38%)	58 (17%)	
Missings		0	4	
**Finds it difficult to wear a mask**	32<br>4			0.13
Yes		5 (56%)	90 (29%)	
No		4 (44%)	225 (71%)	
Missings		4	36	
**Did attend emergency care ("Notbetreuung") during lockdown**	36<br>3			0.5
Yes		4 (31%)	74 (21%)	
No		9 (69%)	276 (79%)	
Missings		0	1	
**Freq. of meetings with children (outside school/kindergarten) since the beginning of the pandemic**	35<br>9			>0.9
> 3 mal		2 (15%)	50 (14%)	
1-2 mal		7 (54%)	203 (59%)	
gar nicht		4 (31%)	93 (27%)	
Missings		0	5	

Children stratified by Euroimmun ® ELISA test result	N	Positive, N = 13[1]	Negative, N = 351[1]	p-value[2]
**Number of other children, when meeting them**	36<br>2			0.6
>5		0 (0%)	10 (2.9%)	
1-2		10 (77%)	204 (58%)	
2-5		0 (0%)	8 (2.3%)	
3-5		0 (0%)	55 (16%)	
Not applicable		3 (23%)	72 (21%)	
Missings		0	2	
**Hugs friends regularly**	35<br>9			0.7
Yes		3 (23%)	63 (18%)	
No		10 (77%)	283 (82%)	
Missings		0	5	
**Wearing a mask when meeting other children**	36<br>4			>0.9
Yes		1 (7.7%)	35 (10.0%)	
No		12 (92%)	299 (85%)	
Not applicable		0 (0%)	17 (4.8%)	
**Meeting with friends less frequently since beginning of the pandemic**	36<br>1			0.7
Yes		11 (85%)	303 (87%)	
No		2 (15%)	45 (13%)	
Missings		0	3	
**Result PCR-test of one Parent in the past**	35<br>9			**<0.001**
Not applicable		2 (15%)	111 (32%)	
Positive		7 (54%)	15 (4.3%)	
Negative		4 (31%)	220 (64%)	
Missings		0	5	

Children stratified by Euroimmun ® ELISA test result	N	Positive, N = 13[1]	Negative, N = 351[1]	p-value[2]
**Number of adults in the household**	361			0.8
>2		3 (25%)	63 (18%)	
1		1 (8.3%)	33 (9.5%)	
2		8 (67%)	253 (72%)	
Missings		1	2	
**Number of children in the household**	362			>0.9
>3		0 (0%)	12 (3.4%)	
1		3 (25%)	72 (21%)	
2		7 (58%)	197 (56%)	
3		2 (17%)	69 (20%)	
Missings		1	1	
**Facility type**	364			0.073
Mixed school type: Elementary and secondary school		3 (23%)	50 (14%)	
Elementary school		3 (23%)	96 (27%)	
Preschool		5 (38%)	59 (17%)	
Secondary		2 (15%)	146 (42%)	

[1]n (%); Median (IQR)

[2]Pearson's Chi-squared test; Fisher's exact test; Wilcoxon rank sum test
