# Supplementary material for: Longitudinal change in SARS-CoV-2 seroprevalence in 3-to 16-year-old children: The Augsburg Plus study
Source: PLoS One. 2022 Aug 11;17(8):e0272874. doi: 10.1371/journal.pone.0272874 (PMC9371315; doi:10.1371/journal.pone.0272874)
Supplement: S3 Table — (PDF) [file pone.0272874.s006.pdf]

| | Euroimmun® IgG BAU/ml | | | Antibodies against SP1 (Roche® ECLIA) |
|---|---|---|---|---|
| **ID** | **Baseline** | **FUP1** | **FUP2** | |
| 329 | 211,4 | 127,9 | 75,5 | positive (FUP1) |
| 102 | 28,8 | 39,7 | 16,8 | positive (FUP1) |
| 284 | 691,2 | 691,2 | 442,9 | positive (Baseline) |
| 289 | 282,2 | 183,7 | 127,9 | positive (Baseline) |
| 302 | 460,8 | 296,6 | No sampling | positive (Baseline) |
| 301 | 691,2 | 285,7 | 222,9 | positive (Baseline) |
| 252 | 691,2 | 691,2 | 599,0 | positive (Baseline) |
| 28 | 121,0 | 121,0 | 74,9 | negative (FUP2) |
| 114 | 305,3 | 271,9 | 247,1 | positive (Baseline) |
| 121 | 351,4 | 180,3 | 153,2 | positive (Baseline) |
| 164 | 691,2 | 450,4 | 303,0 | positive (Baseline) |
| 276 | 472,3 | 342,1 | 315,6 | positive (Baseline) |
| 333 | 691,2 | 452,2 | 189,5 | No venous Blood sampling |
| 344 | 322,0 | 304,1 | 208,5 | positive (Baseline) |
| 54 | 8,6 | 0,0 | 141,1 | positive (FUP2) |
| 53 | 0,0 | 0,0 | 437,2 | positive (FUP2) |
| 155 | 0,0 | 7,5 | 439,5 | positive (FUP2) |
| 228 | 0,0 | 28,1 | 80,6 | negative (FUP2) |

Violet writing: These participants reported having been infected with COVID-19 at baseline (confirmed by PCR-Test).

Blue writing: These participants did NOT report having been infected with COVID-19.

Red writing: Value is to be interpreted as negative
